# DeepKEGG: a multi-omics data integration framework with biological insights for cancer recurrence prediction and biomarker discovery

**DOI:** 10.1093/bib/bbae185

**Published:** 2024-04-27

**Authors:** Wei Lan, Haibo Liao, Qingfeng Chen, Lingzhi Zhu, Yi Pan, Yi-Ping Phoebe Chen

**Affiliations:** Guangxi Key Laboratory of Multimedia Communications and Network Technology, School of Computer, Electronic and Information, Guangxi University, No. 100 Daxue Road, Xixiangtang District, Nanning 530004, China; Guangxi Key Laboratory of Multimedia Communications and Network Technology, School of Computer, Electronic and Information, Guangxi University, No. 100 Daxue Road, Xixiangtang District, Nanning 530004, China; Guangxi Key Laboratory of Multimedia Communications and Network Technology, School of Computer, Electronic and Information, Guangxi University, No. 100 Daxue Road, Xixiangtang District, Nanning 530004, China; School of Computer and Information Science, Hunan Institute of Technology, No. 18 Henghua Road, Zhuhui District， Hengyang 421002, China; School of Computer Science and Control Engineering, Shenzhen Institute of Advanced Technology, Chinese Academy of Sciences, No. 1068 Xueyuan Avenue, Shenzhen University Town, Nanshan District, Shenzhen 518055, China; Department of Computer Science and Information Technology, La Trobe University, Plenty Rd, Bundoora, Melbourne, Victoria 3086, Australia

**Keywords:** cancer recurrence prediction, interpretability of deep learning, self-attention mechanism, multi-omics data integration

## Abstract

Deep learning-based multi-omics data integration methods have the capability to reveal the mechanisms of cancer development, discover cancer biomarkers and identify pathogenic targets. However, current methods ignore the potential correlations between samples in integrating multi-omics data. In addition, providing accurate biological explanations still poses significant challenges due to the complexity of deep learning models. Therefore, there is an urgent need for a deep learning-based multi-omics integration method to explore the potential correlations between samples and provide model interpretability. Herein, we propose a novel interpretable multi-omics data integration method (DeepKEGG) for cancer recurrence prediction and biomarker discovery. In DeepKEGG, a biological hierarchical module is designed for local connections of neuron nodes and model interpretability based on the biological relationship between genes/miRNAs and pathways. In addition, a pathway self-attention module is constructed to explore the correlation between different samples and generate the potential pathway feature representation for enhancing the prediction performance of the model. Lastly, an attribution-based feature importance calculation method is utilized to discover biomarkers related to cancer recurrence and provide a biological interpretation of the model. Experimental results demonstrate that DeepKEGG outperforms other state-of-the-art methods in 5-fold cross validation. Furthermore, case studies also indicate that DeepKEGG serves as an effective tool for biomarker discovery. The code is available at https://github.com/lanbiolab/DeepKEGG.

## INTRODUCTION

In recent years, with the rapid advancement of high-throughput sequencing technologies, multi-omics data (such as genomics, transcriptomics and proteomics) have rapidly accumulated. These multi-omics data bring unprecedented opportunities and challenges to cancer research [1–4]. Furthermore, the availability of large-scale multi-omics databases such as The Cancer Genome Atlas (TCGA) [5], Cancer Cell Line Encyclopedia (CCLE) [6] and Therapeutically Applicable Research to Generate Effective Treatments (TARGET) (https://www.cancer.gov/ccg/research/genome-sequencing/target) has opened up new possibilities for cancer subtype classification [7, 8], biomarker discovery [9, 10], drug development [11, 12], etc. Multi-omics data integration can comprehensively unveil the molecular-level interconnections and mechanisms underlying cancer development. Furthermore, integrating different types of omics data helps to elucidate potential pathogenic changes underlying cancer and identify potential therapeutic targets [13–15].

Early multi-omics integration methods are mainly based on traditional machine learning models, such as Similarity Network Fusion (SNF) [16] and Neighborhood-based Multi-Omics clustering (NEMO) [17]. For example, Speicher et al. [18] proposed a regularized unsupervised multi-kernel learning ensemble method for cancer subtype classification. This method uses a multi-kernel learning dimensionality reduction framework to implement dimensionality reduction and data integration of multi-omics data. Mo et al. [19] proposed a fully Bayesian latent variable method to integrate multi-omics data. This method can capture the intrinsic structure of multiple omics data by utilizing a small number of latent variables to enable the joint dimensionality reduction of omics data. Huang et al. [20] proposed a unified multi-view cancer subtype clustering method based on adaptive graphs and sparse regularized non-negative matrix factorization. This method integrates the local geometric structure of omics data into the consensus matrix learning process and captures the global structure of multiple omics data by using sparse regularization constraints. Wang et al. [16] constructed sample-specific similarity networks for each omics data and learned features of each sub-network by using graph fusion. Then, these sub-features were fused for cancer subtype classification. Wu et al. [21] proposed a multi-omics data clustering method based on a low-rank approximation to identify molecular subtypes of cancer.

In recent years, deep learning techniques have shown excellent performance in dealing with non-linear problems [22]. Many multi-omics integration methods based on deep learning have been proposed for cancer research. For example, Chai et al. [23] utilized denoising autoencoders to integrate multi-omics data and extracted low-dimensional representations for cancer prognosis and biomarker discovery. Gong et al. [24] designed a self-attention mechanism to reduce the dimensionality of omics data and utilized a multi-omics correlation discovery network to learn cross-omics correlations for cancer biomarker discovery. Liu et al. [25] proposed a computational model based on the convolutional autoencoder for cancer subtype classification. Wang et al. [26] used graph neural networks to extract specific representative features from multi-omics data for cancer classification and biomarker discovery. Li et al. [27] constructed similarity networks for samples using cosine similarity and then learned new omics feature representations for cancer subtype classification and analysis based on the fusion of similarity networks. Oh et al. [28] mapped multi-omics data into pathway images based on the biological relationships between genes and pathways, and utilized convolutional neural networks (CNN) to learn potential omics features of pathway images for cancer prediction and diagnosis. Moon et al. [29] proposed a multi-task attention learning algorithm based on multi-omics data for cancer classification and interpretation. Zhang et al. [30] utilized cycle autoencoder with a shared self-expressive layer to learn low-dimensional representations of the omics samples for disease subtypes identification. Yang et al. [31] proposed a novel multi-omics network fusion method for cancer subtype classification. This method constructs an omics affinity matrix based on Gaussian kernel functions and utilizes low-rank subspace representations for network fusion to classify cancer subtypes. Ahmed et al. [32] proposed a generative adversarial network model to integrate omics data, which is able to capture interaction information from interaction network and omics data. Pang et al. [33] proposed a denoising multi-omics integration method for cancer prognosis prediction. This method uses repeated feature sampling method to obtain significant and stable features from omics data and utilizes attention layers to integrate multi-omics data. Ma et al. [34] proposed a computational method for single-cell biological network inference based on heterogeneous graph transformer. This method first integrates single-cell multi-omics data into a heterogeneous graph with cell nodes and gene nodes. Then, it utilizes transformer to learn low-dimensional representations of nodes for constructing gene regulatory networks and gene association networks.

Although these methods have achieved great successes in the integration of multi-omics data, there are still some limitations: (i) some methods often prioritize higher prediction performance, but they lack an explanation of the working mechanism and decision basis of the model. (ii) Some multi-omics data integration methods simply concatenate multi-omics data into the input space, but they usually ignore the inherent correlations between samples.

In this paper, we propose a novel interpretable framework (DeepKEGG) to predict cancer recurrence and identify biomarkers by integrating multi-omics data including the simple nucleotide variation (SNV) data, gene expression (mRNA) data and miRNA expression (miRNA) data. Specifically, DeepKEGG utilizes the biological relationships between genes/miRNAs and KEGG pathways for feature representation learning and model interpretability. Compared with fully connected neural networks, our method can effectively alleviate the ‘curse of dimensionality’ in the multi-omics data integration process and provide the interpretability of the model. Furthermore, a pathway self-attention module is constructed to improve the prediction performance of the model by learning the correlation features between samples. To demonstrate the prediction performance of DeepKEGG, we conduct five 5-fold cross validation experiments on four TCGA cancer datasets and two TARGET cancer datasets. The experimental results show that DeepKEGG outperforms other advanced classification methods in prediction performance. Moreover, the ablation experiments demonstrate the necessity of multi-omics data integration and the effectiveness of potential feature learning of pathways. Finally, case studies also demonstrate the superiority of DeepKEGG in biomarker discovery and model interpretability. The contributions are summarized as follows:

(i) We construct a biological hierarchical module based on prior biological knowledge for local connectivity of neuron nodes and representation learning of pathway features. This construction method can solve the overfitting problem of small sample high-dimensional omics data in deep neural networks. In addition, the biological hierarchical module contributes to the *post hoc* interpretation of the model, which facilitates understanding and inspection of the model.

(ii) We construct three pathway self-attention module for learning potential correlation features between different samples in the pathway feature space to improve the prediction performance of the model.

(iii) We design a pathway contribution assignment method, which first uses backpropagation to calculate the reference gradient of the input feature (genes/miRNAs) as the contribution score of the feature node to the prediction result. Then, the contribution score of the feature node is redistributed to the connected pathways according to its out-degree to evaluate the contribution of the node to the pathway.

(iv) The experimental results of benchmark datasets demonstrate that our method outperforms than other state-of-the-art methods including stochastic gradient descent (SGD), Logistic Regression (LR), Decision Tree (DT), K-Nearest Neighbor (KNN), Support Vector Machine (SVM), MOGONET [26] and PathCNN [28]. Moreover, the case study shows that it serves as an effective tool for biomarker discovery.

(v) Our model can reveal the regulatory relationships between genes/miRNAs and pathways through biological hierarchical module, which can provide more biological insights.

## MATERIALS AND METHODS

### Overview

**Figure 1:**
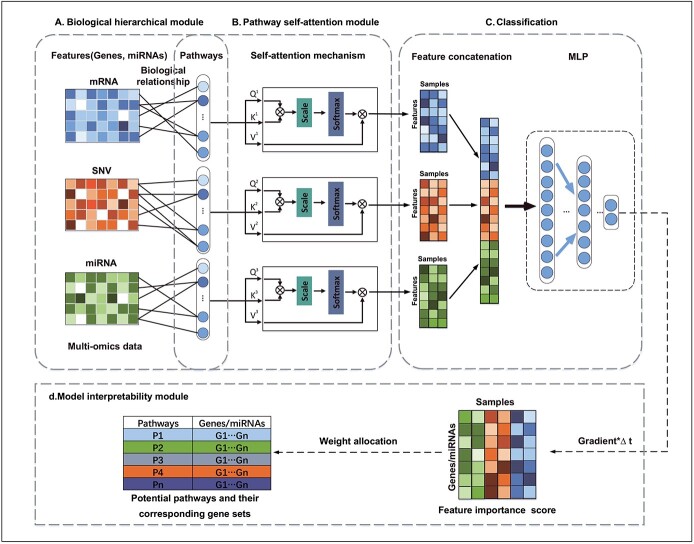
The flowchart of DeepKEGG. (**A**) Biological hierarchical module. (**B**) Pathway self-attention module. (**C**) Classification module. (**D**) Model interpretability module.

The flowchart of DeepKEGG is shown in Figure 1. It mainly consists of four parts: biological hierarchical module, pathway self-attention module, classification module and model interpretability module. First, three relationship matrices (mRNA-pathway, SNV-pathway and miRNA-pathway) are constructed for local connection of neuron nodes and model interpretability based on the gene/miRNA-pathway biological relationships. Then, three pathway self-attention modules are constructed to learn the latent pathway features between different samples. Furthermore, the learned features are concatenated and input into a multi-layer perceptron (MLP) for cancer recurrence prediction. Finally, the attribution-based interpretability method is employed to calculate the importance scores of features for biomarker discovery and model interpretation.

### Data preprocessing

We use cancer datasets from TCGA and TARGET databases to evaluate the prediction performance of our model. First, for the KEGG pathway dataset, the pathway and pathway gene sets are primarily obtained by using the getGenesets of R package EnrichmentBrowser [35]. In addition, the mirPath v.3 web server [36] is used to obtain the miRNA sets of KEGG pathway. The pathway data are preprocessed as follows: (i) pathways associated with specific types of cancer are removed to exclude interference from these specific pathways. (ii) For some pathways with the same name, the detailed names of these duplicated pathways are obtained from the KEGG portal (https://www.kegg.jp/), and name completion is performed for these duplicated pathways. (iii) The gene/miRNA lists on pathways are deduplicated to remove redundant genes/miRNAs.

For the TCGA databases, the clinical data of cancer datasets are downloaded from TCGA including breast cancer (BRCA), liver cancer (LIHC), bladder cancer (BLCA) and prostate cancer (PRAD). Then, we obtain the cancer multi-omics data corresponding to TCGA cancer samples from the Xena TCGA Pan-Cancer web [37] including SNV data, mRNA data (HTSeq-FPKM data type) and miRNA data. Specifically, only samples with matched mRNA, SNV and miRNA data are included in our study. In this experiment, due to the differences in the data format of the raw multi-omics data, it is necessary to perform data preprocessing for each omics data. For mRNA data, the ENSGID identifier is annotated to the corresponding gene name. Only the genes that match the KEGG gene set are retained, while non-coding protein genes are filtered out. In addition, the gene expression values are normalized. For SNV data, the genes with nucleotide variation are set to 1; otherwise, they are set to 0. This operation is repeated for all cancer samples and SNVs. For miRNA data, only the miRNA data matching the miRNA set of KEGG are retained, and the miRNA expression values are normalized. For the TARGET databases, the clinical data, mRNA and miRNA data of Acute Myeloid Leukemia (TARGET-AML) and Wilms Tumor (TARGET-WT) are downloaded from the GDC Data Portal (https://portal.gdc.cancer.gov/v1/). For miRNA data, non-linear transformations are performed using the log2 function and then normalized. The preprocessing of mRNA data is consistent with TCGA. In addition, for repeated samples or genes/miRNAs, the average value of samples or gene/miRNA is taken as the final value. In order to reduce the dimensionality of multi-omics data and eliminate redundant features, the chi-square test is used to feature selection for the multi-omics data. The final details of the omics data for the TCGA datasets and the TARGET datasets are shown in Tables 1 and 2, respectively.

**Table 1:** Summary of the TCGA datasets

Datasets	Categories	Number of features of mRNA, SNV, miRNA	Number of training features of mRNA, SNV, miRNA
PRAD	Recurrence: 100 Non-Recurrence: 150	6037, 4422, 409	1500, 2000, 100
BRCA	Recurrence: 82 Non-Recurrence: 129	6038, 6569, 437	1000, 1000, 100
BLCA	Recurrence: 143 Non-Recurrence: 259	6025, 6819, 479	1000, 1000, 100
LIHC	Recurrence: 171 Non-Recurrence: 183	5823, 5797, 469	1000, 1000, 200

**Table 2:** Summary of the TARGET datasets

Datasets	Categories	Number of features of mRNA, miRNA	Number of training features of mRNA, miRNA
TARGET-AML	Recurrence: 120 Non-Recurrence: 101	6404, 434	2000, 100
TARGET-WT	Recurrence: 88 Non-Recurrence: 24	6758, 441	2000, 200

### Biological hierarchical module

DeepKEGG is designed as a feedforward neural network. The first layer represents the omics feature layer, and the second layer represents the pathway function layer. The first two layers are linked based on the biological relationships between genes/miRNAs and pathways. Let ${X}^z\in{R}^{n\ast k}$ represents the raw input matrix of the *z*-th type of omics data, and ${M}^z\in{R}^{k\ast p}$ represents the relationship matrix between the *z*-th type of omics data and the KEGG pathway. Here, *n* represents the number of samples, *k* represents the number of omics features and *p* represents the number of pathways. The ${M}^z$ is defined as follows: 


(1) \begin{equation*} {M}^z\left(k,p\right)=\left\{\begin{array}{c}\! \ 1, if\ {x}_k^z\ belong\ to\ p\ \\{}0,\ otherwise\end{array}\right. \end{equation*}

where ${x}_k^z$ represents the *k*-th feature of the *z*-th type of omics data, and *p* represents the *p*-th pathway node. If the value of ${M}^z\left(k,p\right)$ is 1, it indicates that the *k*-th feature is connected to the *p*-th pathway node. Otherwise, it is not connected. It should be noted that the relationship matrix construction method between the other omics data and pathways is consistent with it. Then, the propagation from omics feature layer to pathway layer is calculated as follows:


(2) \begin{equation*} {H}^z=h\big({X}^z\ast{M}^z+b\big) \end{equation*}

where ${H}^z$ represents the pathway features of the *z*-th type of omics data, and *b* represents the bias term.

### Pathway self-attention module

In our study, we focus on the correlation between samples, i.e. their proximity in feature space. Typically, samples with similar features exhibit similar behavior or results in the target task. Samples of the same category can improve the representation ability of data by learning the correlation features between each other, and the learned correlation features make the model show better prediction performance. To explore the correlation between different samples, we employ a self-attention mechanism to learn the correlation of pathway features between different samples. We map the pathway feature ${H}^z$ to three different spaces (${Q}^z$, ${K}^z$ and ${V}^z$), i.e. ${Q}^z={H}^z{W}^q,{K}^z={H}^z{W}^k\ and\ {V}^z={H}^z{W}^v$. Let ${H}_i^z$ represents the pathway features of the *i*-th sample of *z*-th type of omics data. The attention score between *i*-th sample and *j*-th sample is defined as follows:


(3) \begin{equation*} {\alpha}_{ij}=\frac{Q_i^z\ast{\left({K}_j^z\right)}^T}{\sqrt{d_k}} \end{equation*}

where ${\alpha}_{ij}$ represents the attention score between *i*-th sample and *j*-th sample, and $\sqrt{d_k}$ represents the scaling factor to prevent gradient explosion. Then, the attention score${\alpha}_{ij}$ is converted to the correlation score ${w}_{ij}$ by using the softmax function, which is defined as follows:


(4) \begin{equation*} {w}_{ij}= Softmax\left({\alpha}_{ij}\right), i\in[1,2,...,n], j\in[1,2,...,n] \end{equation*}

After that the feature matrix ${B}_i^z$ is obtained by weighted summation of the ${V}^z$ matrix and the coefficient score of all sample from one batch, which is defined as follows:


(5) \begin{equation*} {B}_i^z=\sum_j^k{V}_j^z\ast{w}_{ij} \end{equation*}

where ${B}_i^z$ represents the feature matrix of the *i*-th sample of *z*-th type of omics data, and $k$ represents the batch size for model training. The sample correlation construction methods for other omics data are consistent with it. Finally, three feature matrices (mRNA feature matrix, SNV feature matrix and miRNA feature matrix) obtained by the pathway self-attention module are concatenated by a concat function to obtain the final multi-omics feature matrix.


(6) \begin{equation*} Z= concat\left({B}^1,{B}^2,{B}^3\right) \end{equation*}

### Training and optimization

DeepKEGG is a multi-input and single-output end-to-end classification network. In the forward propagation of DeepKEGG, the MLP is used to predict cancer recurrence, which is defined as follows:


(7) \begin{align*}\hat{y_i}=MLP(Z)\end{align*}

In addition, the cross-entropy loss function is used as the objective function, which is defined as follows: 


(8) \begin{align*} Loss= &\frac{1}{N}{\sum}_{i=1}^N\big[-\big({w}_1\ast \big({y}_i\ast \log \big(\hat{y_i}\big)\big)+{w}_0\ast \big(\left(1-{y}_i\right)\nonumber\\&\ast \log \big(1-\hat{y_i}\big)\big)\big)\big]+\gamma{\Vert \alpha \Vert}_2^{2}\end{align*}

(9) \begin{equation*} {w}_j=\frac{N}{n_{class}\ast{N}_j}\ \mathrm{j}\in \left(0,1\right) \qquad\qquad\qquad\qquad\,\quad\qquad\end{equation*}

where ${w}_1$ represents the class weight for positive samples, ${w}_0$ represents the class weight for negative samples. $\gamma$ is the regularization parameter to avoid model overfitting. $\alpha$ denotes all parameters of the model. *N* represents the number of samples, ${n}_{class}$ represents the number of sample classes and ${N}_j$ denotes the number of samples for class *j*.

### Model interpretable module

In order to calculate the impact of genes and miRNA on cancer recurrence, we utilize the DeepLIFT method [38] to compute the importance scores of features (genes, miRNAs). It can calculate the contribution of input features to the prediction results by comparing the differences between the current input and a set of reference inputs. Given a specific cancer sample *S*, the input feature is represented as ${X}^s=\left\{{x}_1^s\dots \dots .{x}_k^s\right\}$ and the final predicted result is represented as y. The contribution score of the input feature is defined as follows:


(10) \begin{equation*} {c}_i^s=\frac{dy}{d{x}_i^s}\Delta t \end{equation*}

where $\frac{dy}{d{x}_i^s}$ represents the gradient of the predicted output *y* with respect to input ${x}_i^s$ and $\Delta t=\left({t}_i-{t}_i^0\right)$ represents the difference between the true activation of current input and the reference activation of the current input.

In this experiment, the value of the reference activation is set to 0. In addition, we sum up the importance scores of all cancer recurrence samples to calculate the overall importance ${C}_i$ of input feature *i*, which is defined as follows:


(11) \begin{equation*} {C}_i=\sum_{s=1}^n{c}_i^s \end{equation*}

The contribution score of pathway nodes is primarily determined by feature nodes. It is calculated based on dividing the total contribution score of the omics feature by its total number of outbound links. Then, the pathway contribution score is obtained by accumulating the average contribution of omics features connected to the same pathway. The contribution score of pathway ${P}_i$ is calculated as follows:


(12) \begin{equation*} mean\_{C}_i=\frac{C_i}{degree\_{out}_i} \end{equation*}

(13) \begin{equation*} {P}_i=\sum_{t=1}^{gn} mean\_{C}_t \end{equation*}

where $degree\_{out}_i$ denotes the out-degree of the *i*-th omics feature. $mean\_{C}_i$ represents the average contribution of the *i*-th omics feature. The $gn$ is the set of omics genes annotated to the *i*-th pathway.

## EXPERIMENTS AND RESULTS

### Evaluation metrics

The accuracy (ACC), precision (PRE), recall (REC), F-score (F1), the area under the receiver operating characteristic curve (AUC) and the area under the precision-recall curve (AUPR) are employed to evaluate the prediction performance of models for cancer recurrence. The ACC is defined as follows:


(14) \begin{equation*} accuracy=\frac{TP+ TN}{TP+ TN+ FP+ FN} \end{equation*}

where TP denotes the number of correctly predicted positive samples by the model, TN denotes the number of correctly predicted negative samples by the model, FP represents the number of negative samples that are incorrectly predicted as positive by the model and FN represents the number of positive samples that are incorrectly predicted as negative by the model.

The precision represents the ratio of true positive predictions to the total number of positive predictions made by the model. The recall represents the ratio of true positive predictions to the total number of actual positive samples in the data. They are defined as follows:


(15) \begin{equation*} precision=\frac{TP}{TP+ FP} \end{equation*}

(16) \begin{equation*} recall=\frac{TP}{TP+ FN} \end{equation*}

The F1-score measures the accuracy of the model that considers both the precision and recall for binary classification tasks, which is the harmonic mean of precision and recall. It is defined as follows:


(17) \begin{equation*} \text{F1-score}=2\ast \frac{Precision\ast Recall}{Precision+ Recall} \end{equation*}

The AUC is the area under the receiver operating characteristic curve. It represents discriminate ability between positive and negative classes at various threshold settings. The AUPR measures the area under the precision–recall curve, which shows the tradeoff between precision and recall for different threshold values.

### Performance comparison with other advanced classification methods on classification tasks

In the experiment, we compare DeepKEGG with seven classification methods including SGD, LR, DT, KNN, SVM, MOGONET [26] and PathCNN [28]. We select these methods for specific reasons: (i) we choose to compare with the interpretable methods DT and PathCNN. The purpose is to prove that our model not only provides better model interpretability (providing information on gene/miRNA-pathway interactions), but also has better prediction performance. (ii) We select five advanced classification methods with SGD, LR, KNN, SVM and MOGONET to further verify the superiority of our proposed model in terms of prediction performance. By comparing with these advanced classification methods, it can better evaluate the performance of our model. For KNN, DT, SVM, LR and SGD methods, the training is performed by directly concatenating omics data as input. For the MOGONET method, we use the preprocessed omics data directly as input. In order to meet the input requirements of the PathCNN method, we use the original omics data as input of the PathCNN. In addition, the default parameters for both methods are used for training. To ensure the stability and accuracy of the prediction results, we conduct five 5-fold cross validation, and take the average of five 5-fold cross validation results as the final prediction results.

**Figure 2:**
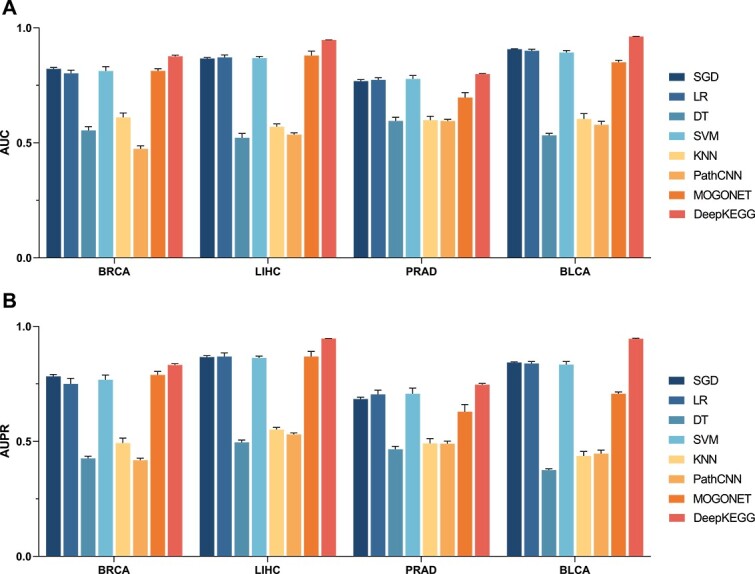
The performance comparison of SGD, LR, DT, SVM, KNN, PathCNN, MOGONET and DeepKEGG in term of AUC and AUPR values on four datasets of TCGA. Performance metrics reported here include: AUC (**A**) and AUPR (**B**).

For the TCGA dataset, the AUC and AUPR of different methods are shown in Figure 2. It can be found that DeepKEGG achieves an average AUC value of 0.876 on the BRCA dataset, which outperforms other methods (SGD: 0.821, LR: 0.802, DT: 0.554, SVM: 0.812, KNN: 0.610, PathCNN: 0.474, MOGONET: 0.812). The average AUC value of DeepKEGG is 0.947 on the LIHC dataset, which surpasses other methods (SGD: 0.867, LR: 0.871, DT: 0.521, SVM: 0.868, KNN: 0.570, PathCNN: 0.535, MOGONET: 0.879). For the PRAD dataset, DeepKEGG achieves an average AUC value of 0.799, which is higher than other methods (SGD: 0.768, LR: 0.773, DT: 0.595, SVM: 0.777, KNN: 0.598, PathCNN: 0.595, MOGONET: 0.697). Lastly, DeepKEGG obtains an average AUC value of 0.961 on the BLCA dataset, which outperforms other methods (SGD: 0.907, LR: 0.900, DT: 0.532, SVM: 0.892, KNN: 0.604, PathCNN: 0.578, MOGONET: 0.850). In addition, the DeepKEGG achieves an average AUPR value of 0.832 on the BRCA dataset, which outperforms other methods (SGD: 0.782, LR: 0.750, DT: 0.426, SVM: 0.768, KNN: 0.492, PathCNN: 0.418, MOGONET: 0.789). For the LIHC dataset, DeepKEGG achieves an average AUPR value of 0.947, which surpasses other methods (SGD: 0.866, LR: 0.869, DT: 0.496, SVM: 0.863, KNN: 0.551, PathCNN: 0.530, MOGONET: 0.869). DeepKEGG achieves an average AUPR value of 0.747 on the PRAD dataset, which outperforms other methods (SGD: 0.684, LR: 0.704, DT: 0.465, SVM: 0.707, KNN: 0.491, PathCNN: 0.490, MOGONET: 0.629). Finally, DeepKEGG achieves an average AUPR value of 0.946 on the BLCA dataset, which outperforms other methods (SGD: 0.843, LR: 0.839, DT: 0.375, SVM: 0.833, KNN: 0.436, PathCNN: 0.446, MOGONET: 0.706). In addition, Table 3 shows the other metrics (ACC, REC and F1-score) for each method on the four TCGA datasets. From the above results, it can be observed that DeepKEGG outperforms other methods on four datasets. In addition, the AUC of DeepKEGG outperforms MOGONET (ranked second in prediction performance) by 6%, and the AUPR outperforms MOGONET by 7% on the LIHC dataset.

**Table 3:** Performance comparison of methods based on 5-fold cross validation on four TCGA datasets

Datasets	Methods	ACC	REC	F1-score
BRCA	SGD	0.738 ± 0.017	0.602 ± 0.067	0.631 ± 0.039
	LR	0.726 ± 0.008	0.501 ± 0.024	0.581 ± 0.015
	DT	0.580 ± 0.015	0.436 ± 0.014	0.443 ± 0.019
	SVM	0.746 ± 0.008	0.511 ± 0.016	0.604 ± 0.015
	KNN	0.615 ± 0.004	0.033 ± 0.014	0.061 ± 0.025
	PathCNN	0.488 ± 0.019	0.391 ± 0.038	0.357 ± 0.038
	MOGONET	0.763 ± 0.016	0.489 ± 0.034	0.596 ± 0.033
	DeepKEGG	0.784 ± 0.006	0.603 ± 0.008	0.672 ± 0.007
LIHC	SGD	0.756 ± 0.014	0.726 ± 0.069	0.740 ± 0.019
	LR	0.777 ± 0.023	0.732 ± 0.020	0.760 ± 0.023
	DT	0.522 ± 0.016	0.517 ± 0.026	0.510 ± 0.021
	SVM	0.783 ± 0.008	0.718 ± 0.017	0.762 ± 0.011
	KNN	0.555 ± 0.012	0.253 ± 0.014	0.352 ± 0.019
	PathCNN	0.521 ± 0.015	0.547 ± 0.033	0.520 ± 0.011
	MOGONET	0.812 ± 0.009	0.852 ± 0.026	0.816 ± 0.011
	DeepKEGG	0.877 ± 0.006	0.856 ± 0.009	0.870 ± 0.007
PRAD	SGD	0.717 ± 0.011	0.606 ± 0.037	0.622 ± 0.018
	LR	0.726 ± 0.006	0.530 ± 0.020	0.602 ± 0.017
	DT	0.613 ± 0.016	0.504 ± 0.018	0.507 ± 0.016
	SVM	0.712 ± 0.008	0.508 ± 0.011	0.581 ± 0.008
	KNN	0.613 ± 0.011	0.086 ± 0.025	0.145 ± 0.040
	PathCNN	0.556 ± 0.018	0.578 ± 0.077	0.486 ± 0.038
	MOGONET	0.647 ± 0.026	0.640 ± 0.030	0.592 ± 0.024
	DeepKEGG	0.736 ± 0.007	0.699 ± 0.016	0.679 ± 0.006
BLCA	SGD	0.799 ± 0.021	0.709 ± 0.061	0.712 ± 0.034
	LR	0.818 ± 0.005	0.668 ± 0.018	0.722 ± 0.011
	DT	0.567 ± 0.011	0.410 ± 0.012	0.399 ± 0.007
	SVM	0.811 ± 0.006	0.634 ± 0.006	0.703 ± 0.005
	KNN	0.621 ± 0.020	0.436 ± 0.080	0.440 ± 0.045
	PathCNN	0.567 ± 0.028	0.440 ± 0.039	0.385 ± 0.026
	MOGONET	0.777 ± 0.007	0.667 ± 0.033	0.681 ± 0.016
	DeepKEGG	0.896 ± 0.003	0.875 ± 0.013	0.857 ± 0.006

*Note:* Result: mean ± standard deviation.

Furthermore, we conduct five 5-fold cross validation to compare DeepKEGG with other methods on TARGET datasets. The results are shown in Table 4. It can be found that DeepKEGG achieves an average AUC value of 0.850 on the TARGET_AML dataset, which outperforms other methods (SGD: 0.836, LR: 0.833, DT: 0.661 SVM: 0.784, KNN: 0.667, PathCNN: 0.578, MOGONET: 0.806). The average AUC value of DeepKEGG is 0.852 on the TARGET_WT dataset, which surpasses other methods (SGD: 0.847, LR: 0.844, DT: 0.505, SVM: 0.832, KNN: 0.732, PathCNN: 0.546, MOGONET: 0.829). In addition, the DeepKEGG achieves an average AUPR value of 0.883 on the TARGET_AML dataset, which outperforms other methods (SGD: 0.858, LR: 0.859, DT: 0.646, SVM: 0.824, KNN: 0.692, PathCNN: 0.661, MOGONET: 0.834). DeepKEGG achieves an average AUPR value of 0.962 on the TARGET_WT dataset, which surpasses other methods (SGD: 0.957, LR: 0.954, DT: 0.789, SVM: 0.953, KNN: 0.882, PathCNN: 0.831, MOGONET: 0.948). It can observe that DeepKEGG performs better on both datasets, indicating the potential applicability of DeepKEGG with a wide range for multi-omics data integration.

**Table 4:** Performance comparison of methods on two datasets of TARGET based on 5-fold cross validation

Datasets	Methods	ACC	PRE	F1-score	AUC	AUPR
TARGET-AML	SGD	0.742 ± 0.012	0.785 ± 0.013	0.751 ± 0.014	0.836 ± 0.001	0.858 ± 0.001
	LR	0.740 ± 0.014	0.769 ± 0.016	0.758 ± 0.012	0.833 ± 0.012	0.859 ± 0.006
	DT	0.667 ± 0.015	0.690 ± 0.005	0.699 ± 0.018	0.661 ± 0.013	0.646 ± 0.009
	SVM	0.716 ± 0.014	0.758 ± 0.019	0.731 ± 0.013	0.784 ± 0.001	0.824 ± 0.004
	KNN	0.633 ± 0.008	0.697 ± 0.021	0.634 ± 0.006	0.667 ± 0.009	0.692 ± 0.007
	PathCNN	0.537 ± 0.018	0.571 ± 0.021	0.567 ± 0.017	0.578 ± 0.004	0.661 ± 0.003
	MOGONET	0.719 ± 0.014	0.772 ± 0.029	0.718 ± 0.004	0.806 ± 0.005	0.834 ± 0.007
	DeepKEGG	0.772 ± 0.005	0.788 ± 0.004	0.792 ± 0.005	0.850 ± 0.001	0.883 ± 0.001
TARGET-WT	SGD	0.818 ± 0.018	0.865 ± 0.016	0.887 ± 0.011	0.847 ± 0.009	0.957 ± 0.002
	LR	0.796 ± 0.016	0.843 ± 0.008	0.875 ± 0.010	0.844 ± 0.004	0.954 ± 0.005
	DT	0.673 ± 0.011	0.788 ± 0.008	0.792 ± 0.008	0.505 ± 0.018	0.789 ± 0.006
	SVM	0.787 ± 0.010	0.842 ± 0.007	0.869 ± 0.007	0.832 ± 0.013	0.953 ± 0.004
	KNN	0.791 ± 0.004	0.800 ± 0.003	0.880 ± 0.002	0.732 ± 0.021	0.882 ± 0.008
	PathCNN	0.586 ± 0.029	0.784 ± 0.004	0.708 ± 0.029	0.546 ± 0.027	0.831 ± 0.009
	MOGONET	0.823 ± 0.008	0.886 ± 0.018	0.888 ± 0.005	0.829 ± 0.021	0.948 ± 0.007
	DeepKEGG	0.830 ± 0.005	0.900 ± 0.006	0.892 ± 0.003	0.852 ± 0.003	0.962 ± 0.001

*Note*: Result: mean ± standard deviation.

The above experiment results demonstrate the effectiveness of DeepKEGG in the integration of multi-omics data by using biological relationships between genes/miRNAs and pathways. It also confirms that DeepKEGG can capture more correlation information by using the pathway self-attention module to improve the predictive capacity of the model. In addition, it can be found that KNN and tree-based models show relatively poor predictive performance. The possible reason may be that these methods are hard to capture effective feature information due to the high-dimensional, sparse and heterogeneous characteristics of multi-omics data. In conclusion, the above experiments confirm the superiority and applicability of DeepKEGG in cancer recurrence prediction.

### Prediction performance of DeepKEGG in multi-omics data integration and single omics data

**Figure 3:**
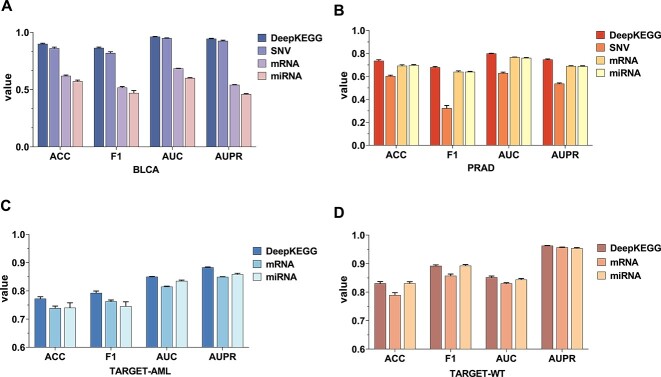
Performance comparison of DeepKEGG on multi-omics data and single-omics data. (**A**) Results of the BLCA dataset. (**B**) Results of the PRAD dataset. (**C**) Results of the TARGET-AML dataset. (**D**) Results of the TARGET-WT dataset.

In order to further demonstrate the necessity of integrating multi-omics data for cancer recurrence prediction, we compare the classification results of integrated multi-omics data and single omics data on four datasets including the BLCA, PRAD, TARGET-AML and TARGET-WT datasets. The results are shown in Figure 3. It can be observed that the integration of multi-omics data outperforms single omics data in predictive performance. This indicates that different types of omics data are complementary, which can provide different levels of biological information. Furthermore, it is observed that the SNV performs better than mRNA and miRNA for the cancer recurrence prediction task on the BLCA dataset. It suggests that SNV has a stronger correlation with the recurrence of BLCA compared with mRNA and miRNA data. The mRNA and miRNA data perform better than SNV for the cancer recurrence prediction task on the PRAD dataset. It shows that mRNA and miRNA play a dominant role in the recurrence of PRAD compared with SNV. Specifically, integrating multiple omics data can compensate for the limitations of single omics data and improve the robustness and predictive ability of models. In addition, DeepKEGG has excellent scalability to accommodate different number of omics data types.

### Performance of DeepKEGG under different hyper-parameter k

**Figure 4:**
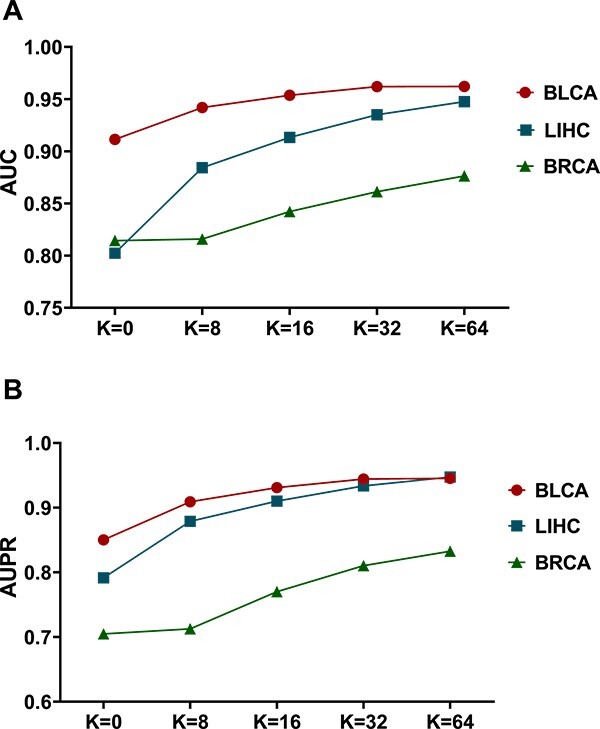
Performance of DeepKEGG under different values of hyper-parameter k. Performance metrics reported here include: AUC (**A**) and AUPR (**B**).

In order to test the effect of parameter k which represents the feature dimension of the output in the pathway self-attention module, we conduct five 5-fold cross validation experiments on three datasets including BLCA, LIHC and BRCA. AUC and AUPR are selected as evaluation metrics. The setting of K should be determined based on the scale of the dataset. When a smaller value is chosen for K, the model may struggle to effectively represent and process the data during training. In contrast, setting a larger value for K can lead to model overfitting, especially in small-sample datasets. Based on the size of our datasets (the cancer dataset samples are between 200 and 400), the value of k is arranged on small sequences [0,8,16,32,64]. It should be noted that the pathway self-attention module is not included when k = 0. The final evaluation results (the average value of the five 5-fold cross validation experiment results) are shown in Figure 4. It is observed that the AUC and AUPR on the three datasets increase with the increase of parameter k. Compared with DeepKEGG without the pathway self-attention module (k = 0), DeepKEGG with the pathway self-attention module achieves better results in the performance metrics. This suggests that the pathway self-attention module can learn correlation features between different samples and helps to improve the prediction performance of the model. In order to obtain the best prediction results, we set k = 64 as the final hyperparameter value of DeepKEGG.

### Identify the potential genes/miRNAs related to cancer recurrence by DeepKEGG

To explore the impact of different omics data on cancer recurrence, we calculate the importance scores for each type of omics data and select the top 20 features (genes or miRNAs) as the biomarkers of cancer recurrence. The higher importance scores indicate that the corresponding features have a stronger impact on cancer recurrence. Table 5 shows the top 20 biomarkers associated with LIHC and BLCA. Then, we validate identified genes or miRNAs related to cancer by consulting a literature review.

**Table 5:** Potential genes/miRNAs identified by DeepKEGG for LIHC and BLCA

Datasets	Omics data type	Top 20 important biomarkers
LIHC	SNV	*ADCY9, IKBKB, CYP2E1, TRAF6, LYN, BCL2, MAP2K1, HSPA6, ACOX3, LAMB1, KRT23, CDKN1B, PTK2, RPS6KB1, AKT2, PARP2, BAAT, PRKACB, VTN, NFATC1.*
	mRNA	*CYP1A2, TAT, ACAT2, ELK1, CXCL12, CPT1B, CYP2E1, EGF, CTNNA3, PIK3R2, IFNG, INPP4A, ACACA, SEC61A2, CD8A, ATP2A3, EFNA3, CDC25B, IPMK, PRF1.*
	miRNA	*hsa-mir-483, hsa-mir-518c, hsa-mir-186, hsa-mir-107, hsa-mir-641, hsa-mir-217, hsa-mir-7-1, hsa-mir-519d, hsa-mir-767, hsa-mir-339, hsa-mir-624, hsa-mir-520a, hsa-mir-1224, hsa-mir-643, hsa-mir-3653, hsa-mir-493, hsa-mir-1295a, hsa-mir-3615, hsa-mir-520b, hsa-mir-376b*.
BLCA	SNV	*GSTT2B, EGF, CACNA1C, ERBB2, ITPR3, VAV1, PPP3CB, PLA2G4F, GRIA4, SKP1, SLC4A4, ABL1, SEC61A1, ITGA3, GSTP1, SIN3A, GRIK3, IL10RB, PGAM2, HSD17B12.*
	mRNA	*ADCY3, PRKAA2, ALDH3A1, ADCY9, CALML3, COL1A1, CHEK1, PLA2G4E, COL6A3, FBP1, FN1, CD3D, MIOX, WNT3A, STXBP1, FGF22, KITLG, IRS1, PIK3CG, NDUFS8.*
	miRNA	*hsa-mir-548y, hsa-mir-199b, hsa-mir-133b, hsa-mir-4691, hsa-mir-577, hsa-mir-494, hsa-mir-615, hsa-mir-432, hsa-mir-455, hsa-mir-665, hsa-mir-4524a, hsa-mir-4733, hsa-mir-377, hsa-mir-658, hsa-mir-1260b, hsa-mir-487a, hsa-mir-506, hsa-mir-380, hsa-mir-152, hsa-mir-485.*

For the LIHC dataset, among the biomarkers identified from the SNV data, it has been proved that low expression of *CYP2E1* may play a crucial role in promoting the malignant progression of hepatocellular carcinoma, which could serve as a potential biomarker for predicting the prognosis of hepatocellular carcinoma [39]. It has been revealed that *TRAF6* may contribute to the metastasis and progression of hepatocellular carcinoma and could serve as a promising target in the treatment of hepatocellular carcinoma [40]. For the biomarkers identified from the mRNA data, *CYP1A2* has been identified as a potential cancer suppressor and an independent prognostic marker for patients with hepatocellular carcinoma [41]. Some studies have demonstrated that *CXCL12* promotes the growth, invasion and angiogenesis of hepatocellular carcinoma, which is considered a potential biomarker for hepatocellular carcinoma [42, 43]. Among the biomarkers identified from the miRNA data, it has been revealed that the overexpression of *has-miR-483* has been proven to promote the proliferation of hepatocellular carcinoma cells, which is a potential specific and sensitive biomarker for the diagnosis of hepatocellular carcinoma [44]. It has been discovered that the *has-miR-186* is regulated by the *SNHG16* gene and is involved in the proliferation, migration and invasion of hepatocellular carcinoma cells [45].

For the BLCA dataset, among the biomarkers identified from the SNV data, it has been revealed that *EGF* promotes bladder cancer cell proliferation via modulation of AR signals [46]. Some studies have shown that high expression of *ERBB2* is an independent risk factor for reduced recurrence-free survival in patients with non-muscle-invasive bladder cancer [47]. In addition, it has been proved that the mutation of *ERBB2* also affects the proliferation and signaling pathways of bladder cancer cells [48]. For the biomarkers identified from the mRNA data, it has been discovered that patients with low expression of *CHEK1* have a higher survival rate in bladder cancer than those with high expression of *CHEK1* [49]. It has been found that *CD3D* is associated with the survival of muscle-invasive bladder cancer, and CD3D/CD4 can serve as independent prognostic factors for muscle-invasive bladder cancer [50]. Among the biomarkers identified from the miRNA data, it has been demonstrated that the *has-miR-432* could target RNA-binding motif protein 5 to regulate apoptosis in bladder cancer cells and high expression of *has-miR-432* is significantly associated with poorer overall survival in bladder cancer patients [51]. It has been demonstrated that *has-miR-665* inhibits the migration of bladder cancer cells by regulating the expression of *SMAD3* and *SNAIL*. It suggested that *has-miR-665* could serve as a therapeutic target for bladder cancer [52].

**Figure 5:**
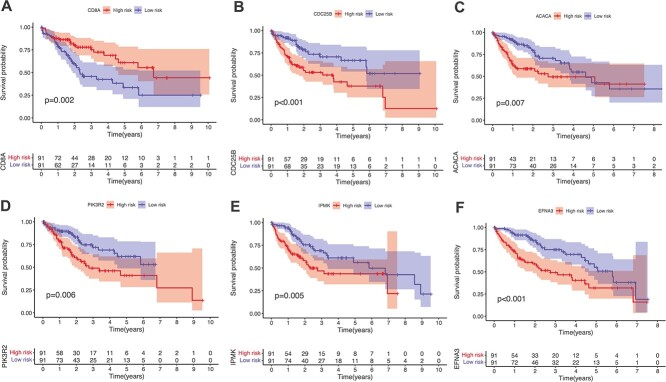
Kaplan–Meier curves of mRNA genes for LIHC. (**A**) Kaplan-Meier curve of the *CD8A *gene. (**B**) Kaplan-Meier curve of the *CDC25B *gene. (**C**) Kaplan-Meier curve of the *ACACA *gene. (**D**) Kaplan-Meier curve of the *PIK3R2 *gene. (**E**) Kaplan-Meier curve of the *IPMK *gene. (**F**) Kaplan-Meier curve of the *EFNA3 *gene.

**Figure 6:**
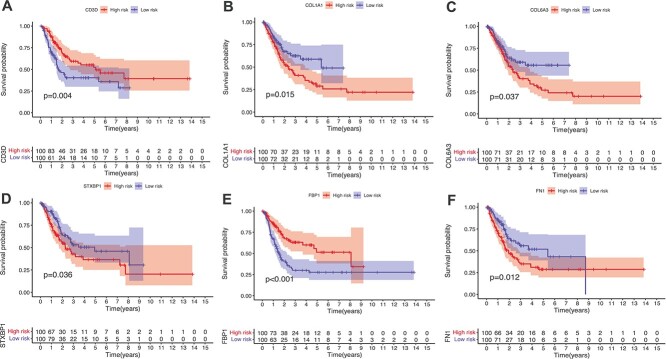
Kaplan–Meier curves of mRNA genes for BLCA. (**A**) Kaplan-Meier curve of the *CD3D *gene. (**B**) Kaplan-Meier curve of the *COL1A1 *gene. (**C**) Kaplan-Meier curve of the *COL6A3 *gene. (**D**) Kaplan-Meier curve of the *STXBP1 *gene. (**E**) Kaplan-Meier curve of the *FBP1 *gene. (**F**) Kaplan-Meier curve of the *FN1 *gene.

Furthermore, we conduct survival analysis on the genes from mRNA data in these two datasets to validate the impact of biomarkers identified by DeepKEGG on patient survival. First, samples are divided into high- and low-risk groups based on the expression values of genes. Samples with expression values in the lower quartile are classified into the low-risk group, while samples with expression values in the upper quartile are classified into the high-risk group. Then, Kaplan–Meier curves are plotted using the ‘survival’ package in R language. The results are shown in Figures 5 and 6. It shows that some of the top-ranking genes show significant differences in survival between the high- and low-risk groups (*P* < 0.05), which indicates the association between the expression levels of these genes and patient survival status. Specifically, the survival analysis results indicate the potential value of the biomarkers identified by DeepKEGG in predicting patient survival. Although other genes/miRNAs are not verified by the current study, it deserves biologists to further study by using experimental method.

#### Biological interpretation of the model

**Figure 7:**
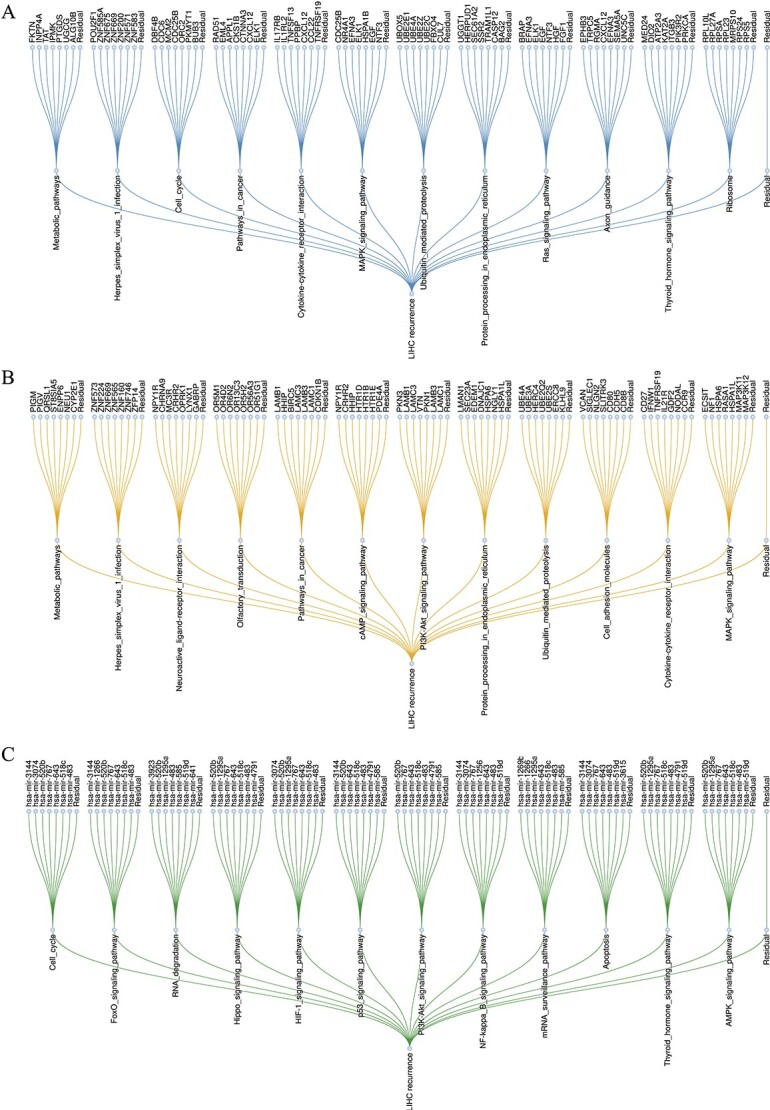
Visualization of node importance for LIHC recurrence. (**A**) Visualization of node importance of mRNA data from LIHC. (**B**) Visualization of node importance of SNV data from LIHC. (**C**) Visualization of node importance of miRNA data from LIHC. This figure shows the pathway nodes and their gene sets that have a large contribution in liver cancer recurrence. For each subgraph from top to bottom, the first layer is the gene/miRNA nodes, which show the seven most important genes/miRNAs in each pathway, while the residual genes/miRNAs are labeled ‘Residual’. The second layer is the pathway nodes, which show the top 12 pathways, while the residual pathways are labeled ‘Residual’. For all nodes, the leftmost node is the node with the highest order of importance, and then its importance decreases from left to right.

**Figure 8:**
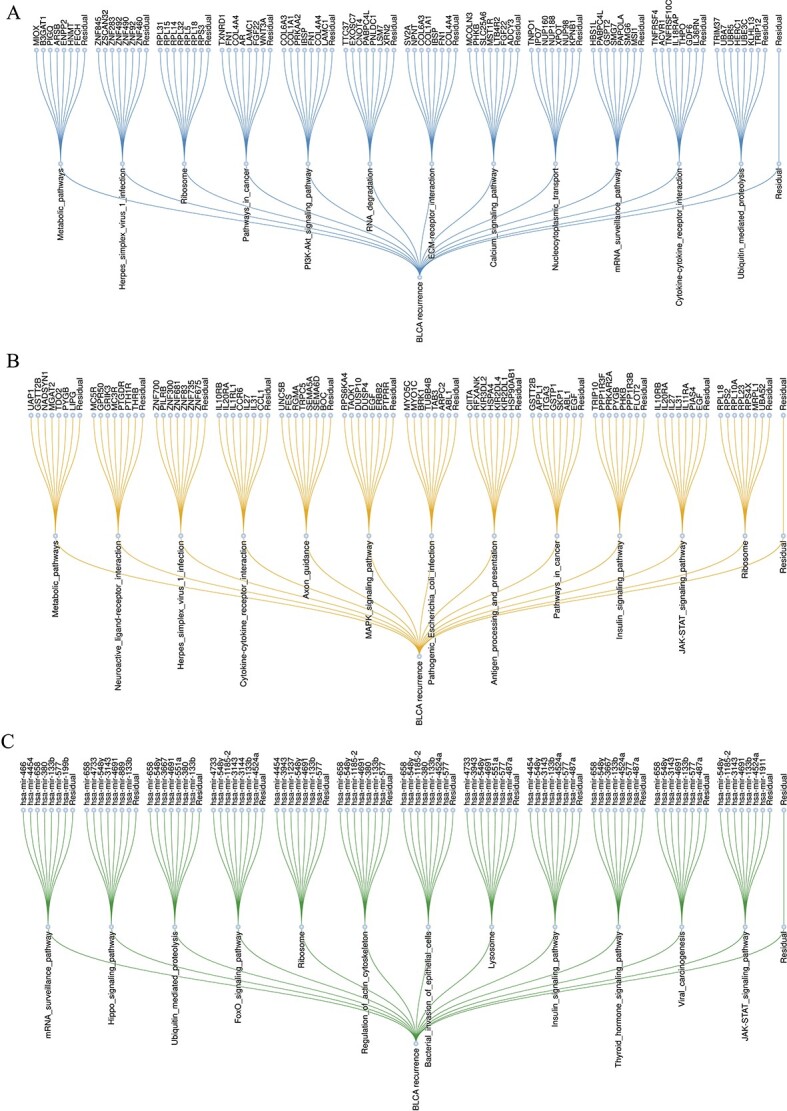
Visualization of node importance for BLCA recurrence. (**A**) Visualization of node importance of mRNA data from BLCA. (**B**) Visualization of node importance of SNV data from BLCA. (**C**) Visualization of node importance of miRNA data from BLCA. This figure shows the pathway nodes and their gene sets that have a large contribution in bladder cancer recurrence. For each subgraph from top to bottom, the first layer is the gene/miRNA nodes, which show the seven most important genes/miRNAs in each pathway, while the residual genes/miRNAs are labeled ‘Residual’. The second layer is the pathway nodes, which show the top 12 pathways, while the residual pathways are labeled ‘Residual’. For all nodes, the leftmost node is the node with the highest order of importance, and then its importance decreases from left to right.

DeepKEGG is able to discover the proteins, pathways and biological processes related to cancer recurrence and provide its quantitative contribution based on the gene layer and pathway layer, which can generate clear and understandable knowledge-based explanations. In addition, DeepKEGG also highlights the gene/miRNA sets of each KEGG pathway, which is involved in the decision-making process. Herein, highly-ranked pathways and their corresponding gene/miRNA sets are selected to provide biological interpretation. Figures 7 and 8 demonstrate the impact of three types of omics data on LIHC and BLCA recurrence, respectively.

For LIHC recurrence, some pathways have been confirmed to be associated with the development of LIHC. For example, it has been verified that the cAMP signaling pathway plays a critical role in the development of liver cancer, which can affect the proliferation, apoptosis and invasion ability of liver cancer cells [53, 54]. In addition, some studies have demonstrated that the degradation of *PDE4A* can activate the cAMP/PKA/CREB signaling pathway and lead to the upregulation of transforming growth factor(TGF)-β expression, which may induce epithelial-mesenchymal transition and promote the invasion of hepatocellular carcinoma [55]. It has been discovered that the overactivation of the MAPK/ERK signaling pathway can enhance the growth and metastatic ability of liver cancer cells, and the MAPK/ERK signaling pathway could be a potential therapeutic target for liver cancer [56]. Furthermore, several studies have shown that the *NTF3* can bind to the p75 neurotrophin receptor and activate the P38 MAPK pathway to promote apoptosis, which may inhibit the development of hepatocellular carcinoma [57]. It has been proved that the Hippo signaling pathway is an important cancer suppressor pathway that plays a crucial role in inhibiting liver cell proliferation, survival and hepatocarcinogenesis [58]. It has been revealed that inhibiting the activation of NF-kappa B can reduce the proliferation and invasive capacity of liver cancer cells and increase their sensitivity to treatment [59]. In addition, some studies have demonstrated that the NF-kB pathway shows constitutive activation in fibrolamellar hepatocellular carcinoma (FLHCC) tissues, which may be involved in the occurrence and development of FLHCC [60].

For BLCA recurrence, some pathways have been confirmed to be associated with the development of BLCA. It has pointed out that Cytokine-cytokine receptor interaction, Neuroactive ligand-receptor interaction and Calcium signaling pathways play important roles in various stages of bladder cancer. In addition, the *COL1A1* and *FN1* genes may affect the progression of bladder cancer by regulating the ECM-receptor interaction [61]. It has been proved that the initiation and progression of bladder cancer are associated with the aberrant activation of the MAPK signaling pathway [62, 63]. Calcium signaling pathway and MAPK signaling pathway are central regulators of bladder cancer development. It has been validated that two pathways can jointly regulate bladder cancer cell cycle arrest and apoptosis [64]. Some studies have demonstrated that the abnormal inactivation of the Hippo signaling pathway is closely associated with the initiation, development and prognosis of bladder cancer [65]. It has revealed that the JAK–STAT signaling pathway is regulated by multiple regulatory factors and involved in the proliferation and migration of bladder cancer cells [66, 67]. Moreover, it has been shown that down-regulation of the activity of the actin cytoskeleton and PI3K-Akt signaling pathway can inhibit the proliferation and migration of bladder cancer [68].

In summary, DeepKEGG is able to identify biological processes and pathways associated with specific cancers. Furthermore, it can also reveal the regulatory relationship between genes/miRNAs and pathways and provide a clear model interpretation.

## CONCLUSION

By integrating data from different omics data such as genomics, transcriptomics and proteomics, deep learning models are able to uncover multi-level cancer characteristics and provide new insights and potential therapeutic approaches. However, there are some challenges to integrating multi-omics data based on deep learning methods, such as how to provide model interpretability and capture the correlation features between different cancer samples. In this paper, we propose an interpretable multi-omics integration method (DeepKEGG) for cancer recurrence prediction and biomarker discovery. DeepKEGG utilizes the biological relationships between genes/miRNAs and pathways for local connectivity. This process not only effectively avoids the excessive number of weights in fully connected neural networks but also enhances the interpretability of the model. In addition, the pathway self-attention module is employed to learn the correlation features of different samples in the pathway feature space to improve the robustness of the model. Finally, important biomarkers are identified by calculating the contribution of the input features (genes and miRNAs) by using the attributional interpretable method. To verify the effectiveness of our model, we compare DeepKEGG with seven state-of-the-art methods (SGD, LR, DT, KNN, SVM, PathCNN and MOGONET) based on 5-fold cross validation. The results further demonstrate the superiority of our model. In addition, ablation experiments demonstrate the importance of multi-omics data integration and the effectiveness of potential feature learning of pathways for cancer recurrence prediction. Furthermore, case studies also indicate that DeepKEGG can serve as an efficient tool for biomarker identification.

Multi-omics data provide information about the molecular characteristics and biomarkers of the tumor, while pathological image data provide information about the morphology, size and boundaries of the tumor. Combining these two types of data can provide more comprehensive and richer tumor feature information, which helps to more accurately assess the cancer status and prognosis of patients. However, dealing with high-dimensional imaging data and providing explanations behind prediction remains a significant challenge [69]. In future work, we will consider integrating pathological image data with omics data and using image interpretability methods to understand the decision-making process of the model and provide more valuable information for clinical diagnosis and treatment [70, 71].

**Key Points**

- We design an interpretable multi-omics data integration framework for cancer recurrence prediction based on the biological relationships between genes/miRNAs and pathways.
- DeepKEGG can efficiently alleviate the curse of dimensionality in the integration of multi-omics data by using the local connectivity of neuron nodes. In addition, it can enhance the prediction performance of the model by employing a pathway self-attention module to learn the correlation features of different samples in the pathway feature space.
- The prediction performance of DeepKEGG on 5-fold cross validation experiments significantly outperforms other classification methods. Furthermore, the case studies suggest that DeepKEGG can serve as an effective tool for biomarker discovery.
- DeepKEGG can reveal the regulatory relationships between genes, pathways and cancers by calculating the contribution scores of biological nodes.

## Data Availability

The codes and datasets are available online at https://github.com/lanbiolab/DeepKEGG.

## Author Biographies

**Wei Lan** received the PhD degree in computer science from Central South University, China, in 2016. Currently, he is the associate professor at School of Computer, Electronic and Information in the Guangxi University, Nanning, China. His current research interests include bioinformatics and machine learning. E-mail: lanwei@gxu.edu.cn

**Haibo Liao** received the BS degree in software engineering from Guangxi University of Science and Technology, China, in 2021. He is currently an MSc student in computer technology, Guangxi University from 2021. His research interests include bioinformatics and deep learning. E-mail: 2113391029@st.gxu.edu.cn

**Qingfeng Chen** received the PhD degree in computer science from the University of Technology Sydney, in 2004. He is currently a professor and the director of the bioinformatics team of the State Key Laboratory for Conservation and Utilization of Subtropical Agro-Bioresources at Guangxi University, China. His research interests include bioinformatics, data mining and artificial intelligence. E-mail: qingfeng@gxu.edu.cn

**Lingzhi Zhu** received the PhD degree in computer science from Central South University, China, in 2022. Currently, he is the associate professor at School of Computer and Information Science in the Hunan Institute of Technology, Hengyang, China. His current research interests include bioinformatics and machine learning. E-mail: zlz@hnit.edu.cn

**Yi Pan** received his PhD degree in computer science from the University of Pittsburgh, USA, in 1991. He is the dean and a professor in the School of Computer Science and Control Engineering, Shenzhen Institute of Advanced Technology, Chinese Academy of Sciences, Shenzhen, Guangdong, China. His current research interests include bioinformatics, big data, machine learning, cloud computing and wireless networks. E-mail: yipan@gsu.edu

**Yi-Ping Phoebe Chen** received the PhD degree in computer science from the University of Queensland. She is a professor and chair of the Department of Computer Science and Computer Engineering at La Trobe University, Australia. Her research interests include bioinformatics, data mining and multimedia. E-mail: phoebe.chen@latrobe.edu.au
